# The First 100 Days of Severe Acute Respiratory Syndrome Coronavirus 2 (SARS-CoV-2) Control in Vietnam

**DOI:** 10.1093/cid/ciaa1130

**Published:** 2020-08-01

**Authors:** Pham Quang Thai, Maia A Rabaa, Duong Huy Luong, Dang Quang Tan, Tran Dai Quang, Ha-Linh Quach, Ngoc-Anh Hoang Thi, Phung Cong Dinh, Ngu Duy Nghia, Tran Anh Tu, La Ngoc Quang, Tran My Phuc, Vinh Chau, Nguyen Cong Khanh, Dang Duc Anh, Tran Nhu Duong, Guy Thwaites, H Rogier van Doorn, Marc Choisy, OUCRU COVID-19 Research Group, Mary Chambers, Marc Choisy, Jeremy Day, Dong Huu Khanh Trinh, Dong Thi Hoai Tam, Joseph Donovan, Du Hong Duc, Ronald B Geskus, Ho Quang Chanh, Hien Ho Van, Huong Dang Thao, Huynh le Anh Huy, Huynh Ngan Ha, Huynh Trung Trieu, Huynh Xuan Yen, Evelyne Kestelyn, Thomas Kesteman, Lam Anh Nguyet, Lam Minh Yen, Katrina Lawson, Le Kim Thanh, Le Nguyen Truc Nhu, Le Thanh Hoang Nhat, Le Thi Hoang Lan, Tan Le Van, Sonia Odette Lewycka, Nguyen Bao Tran, Nguyen Minh Nguyet, Nguyen Than Ha Quyen, Nguyen Thanh Ngoc, Nguyen Thi Han Ny, Nguyen Thi Hong Thuong, Nguyen Thi Huyen Trang, Nguyen Thi Kim Tuyen, Nguyen Thi Ngoc Diep, Nguyen Thi Phuong Dung, Nguyen Thi Tam, Nguyen Thi Thu Hong, Nguyen Thu Trang, Vinh Chau Nguyen Van, Nguyen Xuan Truong, Ninh Thi Thanh Van, Phan Nguyen Quoc Khanh, Phung Khanh Lam, Phung Le Kim Yen, Phung Tran Huy Nhat, Maia Rabaa, Thuong Nguyen Thuy Thuong, Guy Thwaites, Louise Thwaites, Tran My Phuc, Tran Tan Thanh, Tran Thi Bich Ngoc, Tran Tinh Hien, Doorn H Rogier van, Nuil Jennifer van, Vinh Chau, Vu Thi Ngoc Bich, Vu Thi Ty Hang, Sophie Yacoub

**Affiliations:** 1 National Institute of Hygiene and Epidemiology, Hanoi, Vietnam; 2 School of Preventive Medicine and Public Health, Hanoi Medical University, Hanoi, Vietnam; 3 Centre for Tropical Medicine and Global Health, Nuffield Department of Medicine, University of Oxford, Oxford, United Kingdom; 4 Oxford University Clinical Research Unit, Ho Chi Minh city, Vietnam; 5 Medical Services Administration, Ministry of Health, Hanoi, Vietnam; 6 General Department of Preventive Medicine, Ministry of Health, Hanoi, Vietnam; 7 Research School of Population Health, Australian National University, Canberra, Australia; 8 National Agency for Science and Technology Information, Ministry of Science and Technology, Hanoi, Vietnam; 9 Hanoi University of Public Health, Hanoi, Vietnam

**Keywords:** asymptomatic, COVID-19, epidemic control, SARS-CoV-2, Vietnam

## Abstract

**Background:**

One hundred days after severe acute respiratory syndrome coronavirus 2 (SARS-CoV-2) was first reported in Vietnam on 23 January, 270 cases were confirmed, with no deaths. We describe the control measures used by the government and their relationship with imported and domestically acquired case numbers, with the aim of identifying the measures associated with successful SARS-CoV-2 control.

**Methods:**

Clinical and demographic data on the first 270 SARS-CoV-2 infected cases and the timing and nature of government control measures, including numbers of tests and quarantined individuals, were analyzed. Apple and Google mobility data provided proxies for population movement. Serial intervals were calculated from 33 infector-infectee pairs and used to estimate the proportion of presymptomatic transmission events and time-varying reproduction numbers.

**Results:**

A national lockdown was implemented between 1 and 22 April. Around 200 000 people were quarantined and 266 122 reverse transcription polymerase chain reaction (RT-PCR) tests conducted. Population mobility decreased progressively before lockdown. In total, 60% (163/270) of cases were imported; 43% (89/208) of resolved infections remained asymptomatic for the duration of infection. The serial interval was 3.24 days, and 27.5% (95% confidence interval [CI], 15.7%-40.0%) of transmissions occurred presymptomatically. Limited transmission amounted to a maximum reproduction number of 1.15 (95% CI, .·37–2.·36). No community transmission has been detected since 15 April.

**Conclusions:**

Vietnam has controlled SARS-CoV-2 spread through the early introduction of mass communication, meticulous contact tracing with strict quarantine, and international travel restrictions. The value of these interventions is supported by the high proportion of asymptomatic and imported cases, and evidence for substantial presymptomatic transmission.

## Teaser

Vietnam has controlled severe acute respiratory syndrome coronavirus 2 (SARS-CoV-2) spread through early mass communication, contact tracing, quarantine, and travel restrictions. In total, 60% of the 270 cases were imported, 43% were asymptomatic and 27% of transmissions occured before symptoms.

**(See the Editorial Commentary by Cobelens and Harris on pages e343–4.)**

The severe acute respiratory syndrome coronavirus 2 (SARS-CoV-2) emerged in Wuhan city, Hubei Province, China, in late 2019 [1]. On 30 January, the World Health Organization (WHO) declared the outbreak a “Public Health Emergency of International Concern”, and on 11 March a global pandemic. By 1 May 2020, the virus had infected more than 3 million people and killed over 200 000.

SARS-CoV-2 is antigenically different from known human and zoonotic coronaviruses, and there is no known preexisting population immunity [2]. It is highly transmissible through respiratory secretions expelled from an infected person, with a basic reproduction number (R_0_) estimated between 2 and 3 in the absence of control measures [3–6]. Many infections are asymptomatic [7], although others lead to symptoms of coronavirus disease 2019 (COVID-19) of varying severity [5]. Analyses of serial intervals suggest that contagiousness can occur both before and after the onset of symptoms as well as in those who never develop symptoms [8]. The subsequent exponential rise in infections has threatened to overwhelm even the world’s best developed health systems and cause major loss of life. Methods to control the virus and reduce the impact of COVID-19 have thus become a global priority.

The preparedness, timing, and nature of the response to SARS-CoV-2 have varied substantially between countries. Many affected countries have resorted to extreme social distancing measures through so-called lockdowns, where populations isolate themselves within their homes, reducing all but essential contact with others. As first observed in Hubei Province in China, and subsequently in other countries, these measures slow transmission and reduce disease incidence [9–11] but at significant social and economic cost. However, lockdowns represent a combination of potentially independent interventions (eg, closing schools and universities, suspending public transport, banning public gatherings, closing nonessential businesses), the effects of which in isolation are uncertain. Determining their relative contributions to SARS-CoV-2 control is critical to understanding how they might be safely and incrementally lifted or partially reinstated. Such information may be acquired from studying the measures employed by countries that have so far controlled the virus.

Vietnam is a low-middle income country that shares borders with China, The Lao People’s Democratic Republic, and Cambodia. It is the 15th most populous country on earth, with 97.3 million people, and it was one of the first countries affected by SARS-CoV-2, recording its first case on 23 January 2020. Yet by 1 May, 270 cases were confirmed, with no deaths [12]. Here we present a descriptive study that aims to characterize and quantify measures used for SARS-CoV-2 and characteristics of the cases in Vietnam during the first 100 days of the epidemic. Our aim was to identify the measures most closely associated with successful SARS-CoV-2 control.

## METHODS

Clinical, epidemiological, and policy data were provided by Vietnam’s National Steering Committee for COVID-19 response. Data from 270 SARS-CoV-2-confirmed cases to 1 May 2020 included their age, sex, nationality, dates of symptom onset (if any), entry to the country and quarantine (if any), hospital admission and discharge, and the results of reverse transcription polymerase chain reaction (RT-PCR) tests. Imported cases were distinguished from those acquired domestically, with information on quarantine at or after entry to the country. Imported cases were denoted G0; and among domestically acquired infections, those acquired directly from G0 cases were denoted as G1, and others were denoted G2+.

Intervention data consisted of daily time-series of the numbers in quarantine and RT-PCR tests performed. Daily reports from the Ministry of Health and Vietnam’s National Steering Committee for COVID-19 response listed key milestones in national SARS-CoV-2 control measures. Apple mobility data [13] and Google community mobility data [14] provided proxies of population movements, with additional information provided in the Supplementary Appendix.

Serial intervals were calculated from dates of symptoms onset of infector-infectee pairs identified by contact tracing and fitted to a normal distribution by maximum likelihood [8]. The estimated distribution parameters (mean and standard deviation, together with their confidence intervals and variance-covariance matrix) were used to estimate the proportion of presymptomatic transmissions and 3 time-varying reproduction numbers [15]: between G0 and G1 (step 1), between G1 and G2+ (step 2), and between G0, G1, and G2+ combined (step 1 and 2 combined) (further details in the Supplementary Appendix).

We used a logistic regression to investigate the link between the proportion of asymptomatic infections and age, sex, nationality (Vietnamese vs non-Vietnamese), and imported versus domestically acquired infection. We used a gamma regression to investigate the link between the duration of hospitalization and the same variables listed above, plus symptomatic versus asymptomatic. To correct for potential confounding effects between the explanatory variables, we used Type-II likelihood ratio tests [16]. All analyses were done with R 4.0.0 [17] using the packages car [16] 3.0–76, EpiEstim [18] 2.2-1, fitdistrplus [19] 1.0–14, incidence [20] 1.7.17, and mvtnorm [21] 1.1–05, with additional details in the Supplementary Appendix.

## RESULTS

### Epidemic Description and Control Measures

On 10 January, before the first case was confirmed in Vietnam, the Vietnam government reinforced temperature and health status screening at border gates for passengers arriving from Wuhan, tracing and quarantining of suspected cases and their contacts, monitoring of suspected cases of respiratory infections in hospitals and the community, and initiated mass communication to the public on preventive measures (hand washing, contact avoidance, and mask wearing).

**Figure 1.**
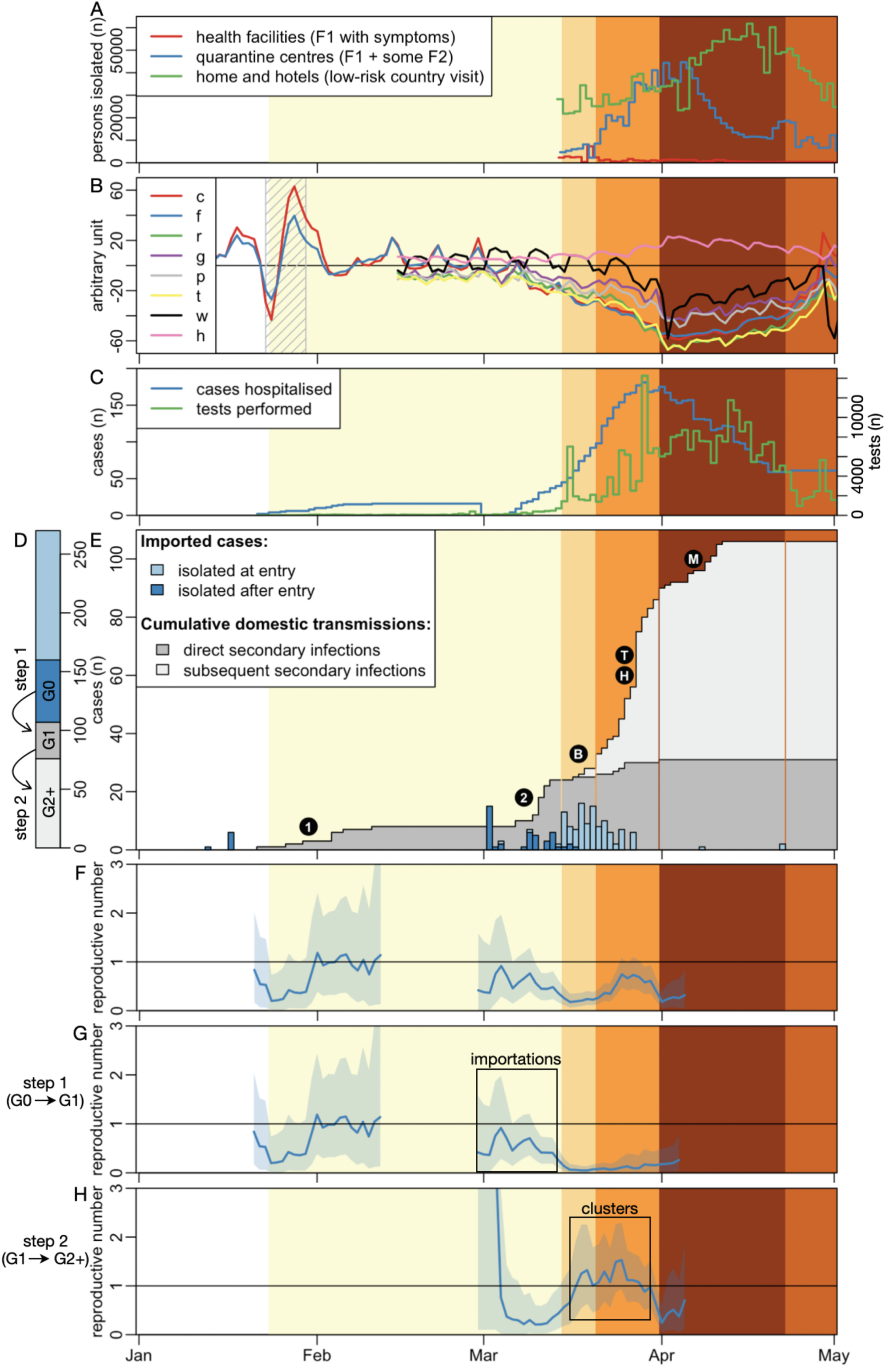
Timeline of SARS-CoV-2 emergence and response in Vietnam. Background color reflects the intensity of the interventions taken by the Vietnam government to control the COVID-19 epidemic, with darker shades indicating more intense disease control measures. The main events of these periods are described in detail in Table 1. *A*, Number of people in isolation by day. *B*, Relative indexes of population movements: number of travellers by car (**c**), on foot (**f**) (both from Apple Mobility Data [13]), proxies of people in retail and recreation areas (**r**), in groceries stores and pharmacies (**g**), in parks (**p**), in bus transit stations (**t**), at work (**w**), and at home (**h**), all from Google Community Mobility Data [14]. Hashed area indicates the lunar New Year holiday (23–29 January). Traditionally, the first half of the week is spent at home with close family, whereas the second half of the week is dedicated to visits of members of the extended family. *C*, Number of SARS-CoV-2 positive cases hospitalised and RT-PCR tests performed by day. *D*, Cumulative number of detected SARS-CoV-2 positive cases in Vietnam, differentiating imported cases (G0) and whether they were isolated at entry or later, and locally transmitted cases and whether they were in direct contact with imported cases (G1) or not (G2+). *E*, Numbers of SARS-CoV-2 imported cases together with cumulative numbers of local transmissions. Circled characters indicate major internal transmission events: first introduction of SARS-CoV-2 virus in the country (**1:** 16 cases), second introduction (**2:** 15 cases), cluster of transmission in a Ho Chi Minh City bar (**B:** 19 cases), cluster of transmission in a large Hanoi hospital (**H:** 17 cases), community cluster of transmission linked to the Hanoi hospital through catering staff (**T:** 28 cases) and community cluster of transmission in Me Linh district in the north of Hanoi (**M:** 13 cases). *F*–*H*, Estimates of the reproduction number for the 2 epidemics. Panel ***G*** focuses only on the first step of the chain of transmission between G0 and G1, whereas panel ***H*** focuses on all the other steps of the chain of transmission. Panel ***F*** includes all detected cases. The shaded blue area shows the 95% confidence intervals. Abbreviations: COVID-19, coronavirus disease 2019; RT-PCR, reverse transcription polymerase chain reaction; SARS-COV-2, severe acute respiratory syndrome coronavirus 2.

The epidemic timeline for Vietnam, including the numbers quarantined and hospitalised, tests performed, cases confirmed, population movements, and the timing and nature of major government-led control measures are summarized in Figure 1. The control measures are summarised in Table 1 and Table S1. To date, 2 waves of transmission have occurred: the first began on 23 January and resulted in 16 cases (9 imported, 7 acquired in-country), and the second on 6 March, leading to 254 cases (154 imported, 100 acquired in-country).

**Table 1.** Timing and Nature of Major Vietnam Government-led Control Measures, Including International Border Control, Internal Control, and Ministry of Health-led Communications

Control measures at international borders		
Phase	Date	Event
	3 January	Strengthening of border control measures announced by the government
	22 January	Monitoring of body temperature and health status at border gates; early case detection and contact tracing with mandatory quarantine started
	28 January to 5 February	Suspension of all flights from China; suspension of tourist visas to foreigners who have been in China; enhanced control of Vietnam-China border; 14-day mandatory quarantine for all travelers who have come from COVID-19 affected areas in China
	23–28 February	Medical declarations for all incoming visitors from Korea; all flights from affected zones diverted to secondary airports outside of HCMC and Hanoi
	28 February	Mandatory 14-day quarantine for all travelers entering Vietnam from a COVID-19 affected country
	15–18 March	Visa suspension for all non-Vietnamese citizens for at least 30 days
	21–22 March	Mandatory 14-day quarantine at centralized facilities for all arriving travelers, regardless of origin; suspension of entry to all foreigners (except for diplomatic and official purposes)
	21–23 March	Vietnam Airlines suspends routes with Singapore, Thailand, Indonesia, Laos, Myanmar, UK, and Japan
	27 March	All individuals entering the country from March 8 onward required to declare and update their health status to aid surveillance; strict control of all entrants by road, sea and air, especially shared borders with Laos and Cambodia
	1 April	Closure of main and auxiliary border gates
**Internal control measures**		
**Phase**	**Date**	**Event**
	20 January	22 hospitals chosen for the treatment of suspected COVID-19
	30 January to 4 May	All schools and universities closed following lunar New Year holiday
	Late January to early February	Field hospitals and quarantine centres established in major cities and near border crossings
	2–16 February	Announcement that the Vietnam Social Insurance health fund will cover SARS-CoV-2 tests and treatment; commune in Vinh Phuc province, Hanoi (10 600 people) quarantined; accelerated domestic production and supply of PPE
	16–20 March	Enforcement of mask wearing at public places; crowds over 50 people discouraged; entertainment services closed
	21 March 21	Mandatory Health Declaration required for passengers on domestic flights and trains; religious services suspended
	25 March 25 to 22 April	Amusement parks, restaurants, catering businesses, billiard clubs, gyms, spas, hair salons close in Ho Chi Minh City
	1–22 April	Declaration of COVID-19 epidemic in Vietnam. Country-wide lockdown implemented; mandatory mask-wearing in public; banning of public gatherings of >2 people; nonessential movement outside of residence discouraged; public transportation and taxi services halted
	23 April	Lockdown measures relaxed, some nonessential businesses remain closed; increased frequency of domestic flights
	4–11 May	Staggered reopening of schools and universities
**Ministry of Health-led Communications**		
**Phase**	**Date**	**Event**
	9 January onward	Dissemination of information advising on the disease situation in China and to maintain calm
	20 January	Updated information concerning the epidemic and case numbers provided every 2 hours on MoH websites ncov.moh.gov.vn and ncov.vncdc.gov.vn
	27 January	Telephone hotline number announced to receive information and opinions on the epidemic and to advise on personal disease prevention
	2 February	Technology-based communication plan established to inform population: SMS to all mobile subscribers; videos and infographics disseminated through mass media, social networks, digital platforms such as Facebook, Zalo, YouTube
	8 February	Vietnam Health App and website launched by MoH to provide information on COVID-19 and disease prevention for the people and healthcare workers
	14 February	Announcement and education around 14-day isolation period for COVID-19 cases and contacts; coordinate with Vietnam Television (VTV) to enhance education and messaging
	23 February	Release of pop song, Ghen Cô Vy (English: Jealous Coronavirus), to promote handwashing, social distancing, not touching one’s face, and keeping their environment clean
	2 March	MoH coordinates with Vietnam Television (VTV 24) to develop daily broadcast on the COVID-19 epidemic
	19 March	Mandatory use of the Hanoi Smart City app to monitor the health and movement of recovered confirmed cases, suspected cases, and people under quarantine
	22 March	Recommendation that people over 60 years stay at home; recommendation that everyone wear a mask when outside of the home and practice good hygiene
	18 April	Release of Bluezone mobile application that uses BLE low-power Bluetooth positioning technology to identify and track and communicate with F1 and F2 contacts when positive cases are detected

Further details are provided in Table S1. The colors shown in the phase column indicate the intensity of control measures taken over different periods (white, initial; light yellow, early; light orange, intermediate; orange, preepidemic; brown, epidemic/lockdown; dark orange, postlockdown), and correspond to those used in Figure 1 and Table S1.

Abbreviations: COVID-19, coronavirus disease 2019; MoH, Ministry of Health; PPE, personal protective equipment; SARS-CoV-2, severe acute respiratory syndrome coronavirus 2.

The first confirmed cases of SARS-CoV-2 infection presented in Hanoi and Ho Chi Minh City during the lunar New Year holiday (23–9 January). Cases were travellers from Wuhan city or their contacts and were identified by the public health laboratory network using improvised molecular diagnostics, including agnostic sequencing, prior to implementation of the World Health Organization (WHO)-approved assays [22]. Among the cases were the first confirmed human-to-human transmissions outside of China [23].

Entry of airline passengers into Vietnam from Wuhan city and elsewhere in China was monitored and progressively limited (Table 1), and cases and their contacts were quarantined for 14 days in government facilities to prevent onward transmission. Schools and universities remained closed after the lunar New Year holiday, with staggered reopening from 4 May (closures lasted ~3 months). The National Steering Committee for COVID-19 response was established in late January, composed of 24 members from 23 ministries charged with coordinating the epidemic response. A hotline was set up by the Ministry of Health on 27 January, a nationwide SMS push notification system was put in place through all mobile phone providers on 3 February, and a mobile phone app for contact tracing and symptom reporting was launched on 8 February.

In early February, following the repatriation of a number of Vietnamese nationals from Wuhan city, a cluster of community transmitted infections was detected in 2 communes in Vinh Phuc province, bordering Hanoi [24, 25]. On 13 February, these communes were quarantined for 3 weeks, with no additional cases detected in the country until 6 March and the start of the second wave of infections in Hanoi.

This second wave began on 6 March following diagnosis of the index case, who had arrived in Hanoi on 2 March from London after visiting Italy and the United Kingdom. Following their identification, all passengers and crew on the flight from London with the index case were quarantined in government facilities for 14 days, as were all individuals in direct contact with the index or any subsequent cases. The immediate neighborhood of the index case was sealed off, with active surveillance conducted to detect any new cases. These surveillance measures revealed SARS-CoV-2 infection in 12 others on the flight and 2 close contacts of the infected traveller after entering Vietnam.

Further cases occurred in the following 2 weeks, mostly in foreign and returning Vietnamese travellers from Europe and the United States, including multiple acquisitions in a Ho Chi Minh City bar on 14 March (19 cases), a cluster among nursing (17 cases) and catering (28 cases) staff in a large Hanoi hospital, and a community cluster in Me Linh district (13 cases), in the north of Hanoi. Systematic layered testing and quarantine requirements were put in place for cases (F0) and their direct (F1) and indirect (F2–4) contacts. Cases were isolated in assigned hospitals until tested negative at least twice by RT-PCR. F1 and F2 contacts were quarantined for at least 14 days in dedicated facilities (health centers, hotels, military camps) with negative tests required before release. F3 and F4 contacts were asked to self-quarantine for 14 days. Until 1 May, around 70 000 have been quarantined in government facilities and around 140 000 at home or in hotels. In total, 266 122 RT-PCR-based SARS-CoV-2 tests were performed, with a ratio of around 1 positive person: 1000 tests conducted.

After further measures to prevent entry of infected international travellers (Table 1), a nationwide lockdown was enforced on 1 April, including closure of all shops except gas stations, food stores, and pharmacies; suspension of public transport, including all taxis; and mandatory mask wearing in all public spaces. Mobility data show that population movement decreased substantially after the start of the second infection wave in early March, reaching a nadir in early April at the start of the lockdown (Figure 1*B*). Movements increased slowly during the last week of the lockdown and more rapidly once the lockdown was partially lifted on 23 April. On 15 April, the last case of wave 2 was identified; subsequent cases (n = 2) have been detected between 15 April and 1 May (time of writing) among international travellers quarantined on arrival.

### Characteristics of the Cases

**Figure 2.**
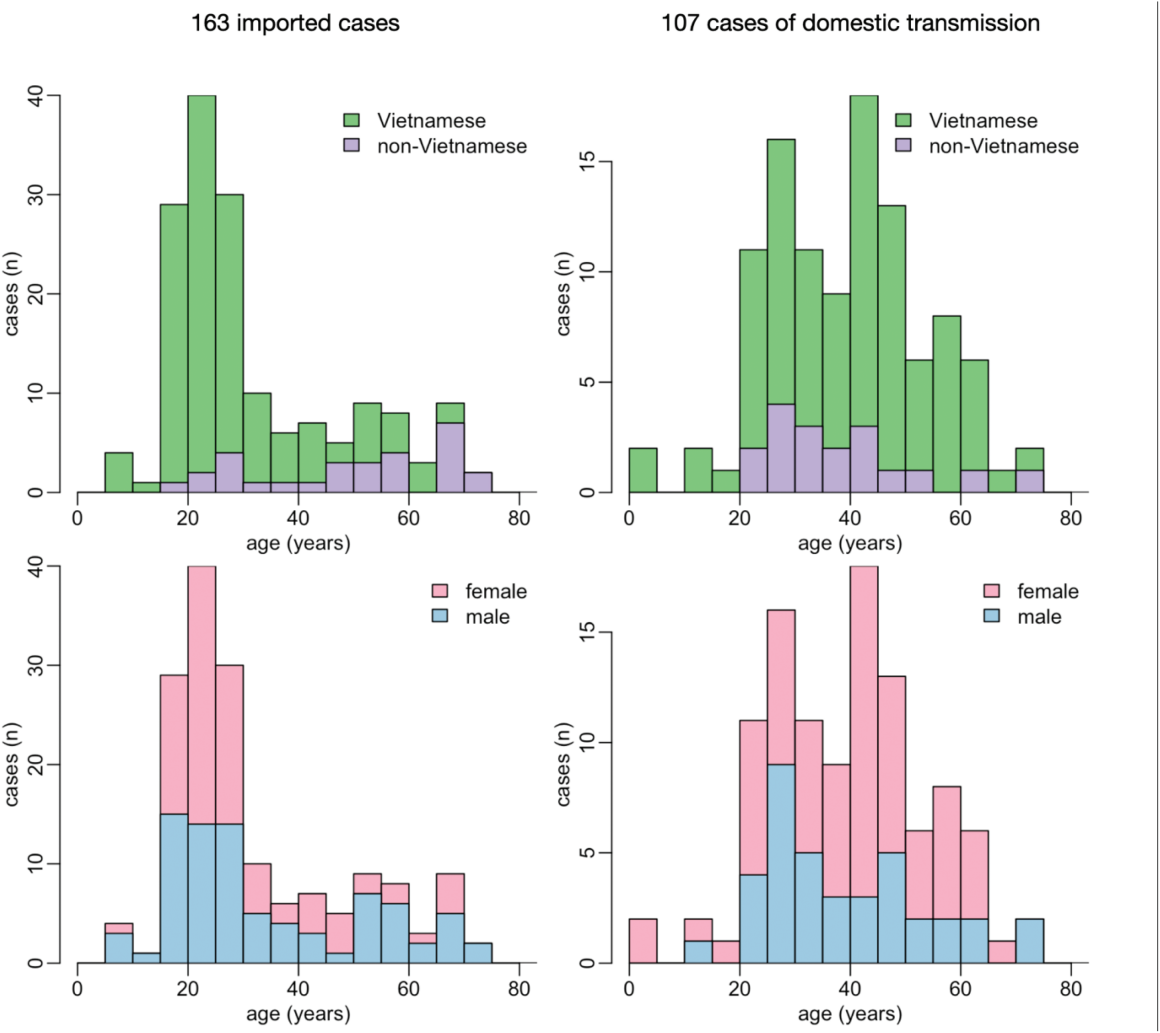
Demographics of the 270 SARS-CoV-2 positive cases in Vietnam. Age distribution for the 163 imported cases (*left column*) and the 107 cases of local transmission (*right column*), by nationality (*top row*) and sex (*bottom row*). Abbreviation: SARS-CoV-2, severe acute respiratory syndrome coronavirus 2.

Sixty percent (163/270) of cases were imported (Table 2, Figure 2); 110 were quarantined and tested positive on entry, whereas 53 entered prior to the implementation of systematic quarantine measures and were identified in the community. Vietnamese nationals represented 134/163 (82.2%) of the imported cases and 89/107 (83.2%) of those acquired in-country. The median age of imported and domestically acquired cases was 27 years (interquartile range [IQR] 21–42) and 41 years (IQR 28–49), and 81/163 (49.7%) and 69/107 (64.5%) of these were female, respectively.

**Table 2.** Clinical and Demographic Characteristics of SARS-CoV-2 Patients

	Asymptomatics	Symptomatics
N	120	150
Age (years)	31 (IQR: 23–45)	30 (IQR: 24–49)
Proportion females	54.2%	56.7%
Proportion G0	57.5%	62.7%
Proportion G1	6.7%	15.3%
Proportions G2+	35.8%	22.0%
Proportion Vietnamese	82.5%	82.7%
Hospitalization duration (days)^a^	17 (IQR: 13–22)	19 (IQR: 16–25)
Proportion in quarantine on arrival^b^	44.2%	38.0%

Abbreviations: IQR, interquartile range; SARS-CoV-2, severe acute respiratory syndrome coronavirus 2.

^a^For those discharged only (ie, n = 89 for the asymptomatics and n = 119 for the symptomatic.

^b^For G0 only.

**Figure 3.**
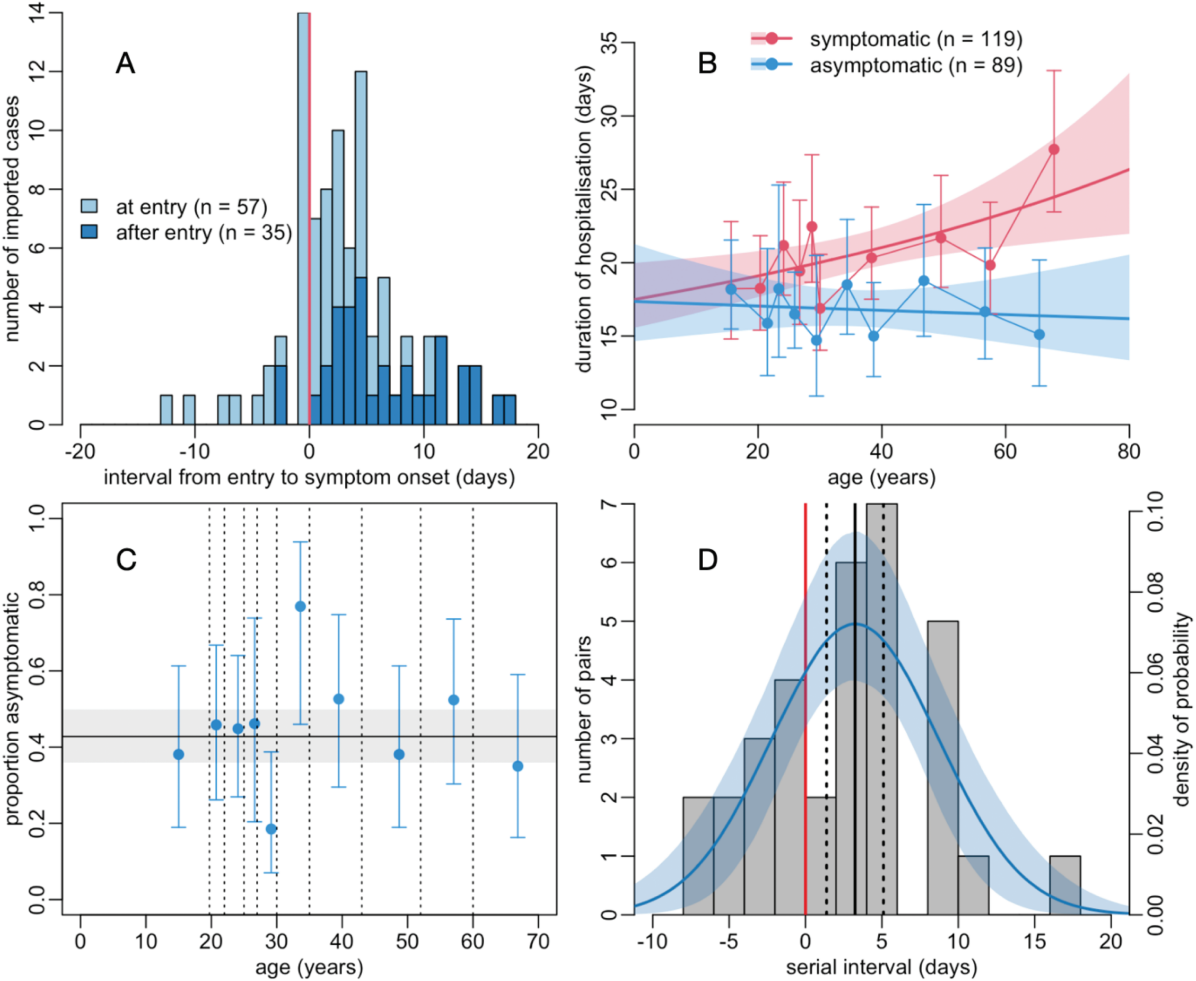
Asymptomatic and symptomatic SARS-CoV-2 infection in Vietnam. *A*, Distribution of the interval between entry into the country and the onset of symptoms for 92 symptomatic imported SARS-CoV-2 positive cases, differentiating those who were isolated at entry from those who were not. Symptoms occurred after entry on the right-hand side of the vertical red line. *B*, Duration of hospital stay of 208 discharged SARS-CoV-2 positive cases. Dots and error bars show mean and 95% confidence interval (assuming a gamma distribution) per decile of age, lines and shaded areas show gamma regression fits and their 95% confidence intervals. Corresponding gamma regression table is in Table S2. *C*, Relationship between age and the proportion asymptomatic among 208 discharged SARS-CoV-2 positive cases. Vertical dotted lines indicate deciles of the age distribution, with the proportion asymptomatic estimated within each of these deciles. Vertical error bars show 95% confidence intervals. Horizontal line and the gray area show the average across ages and its 95% confidence interval. Corresponding logistic regression table is in Table S3. *D*, Distribution of serial intervals for 33 infector-infectee pairs together with a normal distribution fitted to it. The shaded area shows the 95% confidence interval. The vertical black line shows the estimate of the mean serial interval, together with its 95% confidence interval (*dashed vertical lines*). The proportion of the distribution to the left of the red line is a proxy for the proportion of infections that occur before the onset of symptoms. Abbreviation: SARS-CoV-2, severe acute respiratory syndrome coronavirus 2.

By 1 May, 208 patients were discharged, and 62 remained hospitalized for treatment or isolated. Forty-three percent (89/208) of discharged cases never developed symptoms, and this was not significantly associated with age, sex, nationality, or origin of infection (imported or domestically acquired). Among all the symptomatic cases, 25.3% (38/150) developed symptoms in a government quarantine facility. Among the imported cases who developed symptoms, 73.9% (68/92) did so after entry to the country (Figure 3*A*, see Table S4 for the numbers of symptomatic in imported and nonimported cases). The median age of symptomatic and asymptomatic cases was 30 (IQR 24–49) and 31 (IQR 23–45), respectively (no significant effect of age on the probability to develop symptoms, Figure 3*C*). Among the 150 with symptoms, 21 (14.0%) developed severe disease, of whom 5 required mechanical ventilation and 2 received extracorporeal membrane oxygenation. No fatalities were recorded. The duration of hospitalization was significantly shorter (*P* < .0001) for asymptomatic (17 days, IQR 13–22) than for symptomatic cases (19 days, IQR 16–25). Although sex, nationality, and origin of infection did not have any significant effect, the duration of hospitalization of symptomatic cases increased with age (with a discharge rate decreasing by 1.24% for every year older, *P* = .0060) (Figure 3B).

### Epidemiological Parameters Over Time

From 33 infector-infectee pairs, the mean serial interval was estimated to be 3.24 days (95% confidence interval [CI], 1.38–5.10 days) with a standard deviation of the distribution of 5.46 days (95% CI, 4.14–6.78 days). An estimated 27.5% (95% CI, 15.7%–40.0%) of the distribution was below zero, suggesting these transmissions occurred prior to the onset of symptoms in the infector (Figure 3D). From the (nonquarantined) imported cases (G0) and onward infected cases (G1 and G2+), we calculated the effective reproductive number R by date (Figure 1*F–H*). Limited transmission amounted to a maximum R of 1.15 (95% CI, .37–2.36). R rarely exceeded 1, and a decrease of R is seen as more mitigating measures were implemented from the end of March before the nationwide lockdown. When analyzing R from G0 to G1 (step 1) and from G1 to G2+ (step 2) separately, we found that R was drastically decreased for step 1 simultaneously with suspension of all international travel (18 March), whereas for step 2, transmission continues with R slightly above 1 despite intense contact tracing and quarantine. Only during the nationwide lockdown R was reduced to <1 (Figure 1*F* and 1*G*).

## DISCUSSION

On 23 January 2020, Vietnam was one of the first countries to report SARS-CoV-2 infection and the first to report human-to-human transmission outside of China [23]. Yet 100 days later, it confirmed just 270 cases despite extensive testing, with no community transmission since 15 April. In the 3 weeks prior to 1 May, there were only 2 imported cases and no reported cases elsewhere in the country. The nature, timing, and success of the control measures introduced may have relevance to other countries seeking to control SARS-CoV-2 transmission.

Vietnam has experience in responding to emerging infectious diseases. In the last 20 years, it has confronted outbreaks of SARS [26], avian and pandemic influenza [27, 28], hand-foot-and-mouth disease [29], measles [30], and dengue [31]. Its outbreak responses are coordinated by the Ministry of Health, a permanent national Public Health Emergency Operations Center at the National Institute for Hygiene and Epidemiology, and through a network of provincial Centers for Disease Control and lower level preventive medicine centers [32].

Two waves of SARS-CoV-2 infections have occurred over the last 100 days in Vietnam, with community transmission actively interrupted by rapid isolation and identification of primary and secondary cases and their contacts. Around 200 000 people spent at least 14 days in quarantine. Among those quarantined, many were second degree contacts (F2); to our knowledge, no other country has implemented quarantine in this manner. In total, 266 122 RT-PCR tests were performed, primarily in those quarantined, giving a ratio of tests conducted per positive person (~1000:1) or, equivalently, about 200 tested people per positive case.

The majority of cases (60%) in Vietnam were imported from COVID-19 affected countries: first from China and then from Europe and the United States. Early introduction of airport screening, followed by quarantine of all arrivals and the eventual suspension of nearly all international flights prevented further introductions, allowing greater focus on the detection and prevention of domestic transmission. Consistent government communication of disease risk and prevention strategies from 3 February may have contributed to declines in population movement prior to the nationwide lockdown, particularly in March, when all mobile phone users received 10 SMS push notifications from the Ministry of Health in addition to information provided through other media; these early reductions in population movement may have contributed to lowering the reproduction number. The majority of imported cases were <30 years old, and most of those that acquired the infection domestically were <40 years, which may explain the low numbers with severe disease and absence of deaths.

The high proportion of cases that developed symptoms after isolation (73.9%) or never developed symptoms (43%) highlights one of the major challenges of controlling SARS-CoV-2 and the strengths of Vietnam’s approach. Suspected cases were identified and quarantined based on their epidemiological risk of infection (recent contact with a confirmed case or travel to a COVID-19 affected country), rather than on exhibiting symptoms. Without the implementation of strong control measures and meticulous contact tracing, it is likely such cases would have silently transmitted the virus and undermined other control efforts.

The strength of our report is that it provides a complete picture based on national data of case numbers, their clinical and demographic characteristics, and the testing performed and various interventions made by the government over time. Furthermore, the use of systematic quarantine measures allowed clear distinction between imported and domestically acquired cases, thus allowing for estimation of the efficiency of various interventions. The limitations are that the data are descriptive, contain relatively small numbers of confirmed cases, and only include the first 100 days of an epidemic that is likely to continue for many months. It is therefore impossible to conclude definitively which of these control measures have resulted in the current control of SARS-CoV-2 in Vietnam and whether they will continue to work in the future.

There are, however, 2 distinctive features of Vietnam’s response. First, the government acted quickly, educating and engaging the public, placing restrictions on international flights, closing schools and universities, and instituting exhaustive case-contact tracing from late January, well before these measures were advised by WHO. Second, they placed the identification, serial testing, and minimum 14-day isolation of all direct contacts of cases, regardless of symptom development, at the heart of the response. Our findings suggest the latter measure was likely to be especially effective given nearly half of those infected did not develop symptoms.

In summary, Vietnam controlled SARS-CoV-2 spread by acting early, maintaining clear and consistent public communications, introducing meticulous contact tracing and quarantine, and implementing progressive international travel restrictions. The value of these interventions in controlling the infection is supported by the high proportion of asymptomatic cases and imported cases, and evidence for substantial presymptomatic transmission.

## EPILOGUE

There has been no case of community transmission during the 99 days between 16 April and 24 July. Lockdown measures have been progressively lifted and schools, universities, nonessential shops, karaoke bars, and places for mass gatherings have reopened. An additional 146 cases have been confirmed on arrival among repatriated Vietnamese nationals, and they have subsequently been isolated. Over the 5 days before submission (27 July 2020), 14 new cases of community transmission of unknown origin have been detected in the fifth largest city in Vietnam, bringing the total number of cases to 431 and sparking another large public health response.

## Supplementary Data

Supplementary materials are available at *Clinical Infectious Diseases* online. Consisting of data provided by the authors to benefit the reader, the posted materials are not copyedited and are the sole responsibility of the authors, so questions or comments should be addressed to the corresponding author.

ciaa1130_suppl_Supplementary_Appendix

## Notes

OUCRU COVID-19 Research Group Members:

Mary Chambers, Marc Choisy, Jeremy Day, Dong Huu Khanh Trinh, Dong Thi Hoai Tam, Joseph Donovan, Du Hong Duc, Ronald B Geskus, Ho Quang Chanh, Ho Van Hien, Huong Dang Thao, Huynh le Anh Huy, Huynh Ngan Ha, Huynh Trung Trieu, Huynh Xuan Yen, Evelyne Kestelyn, Thomas Kesteman, Lam Anh Nguyet, Lam Minh Yen, Katrina Lawson, Le Kim Thanh, Le Nguyen Truc Nhu, Le Thanh Hoang Nhat, Le Thi Hoang Lan, Le Van Tan, Sonia Odette Lewycka, Nguyen Bao Tran, Nguyen Minh Nguyet, Nguyen Than Ha Quyen, Nguyen Thanh Ngoc, Nguyen Thi Han Ny, Nguyen Thi Hong Thuong, Nguyen Thi Huyen Trang, Nguyen Thi Kim Tuyen, Nguyen Thi Ngoc Diep, Nguyen Thi Phuong Dung, Nguyen Thi Tam, Nguyen Thi Thu Hong, Nguyen Thu Trang, Nguyen Van Vinh Chau, Nguyen Xuan Truong, Ninh Thi Thanh Van, Phan Nguyen Quoc Khanh, Phung Khanh Lam, Phung Le Kim Yen, Phung Tran Huy Nhat, Maia Rabaa, Thuong Nguyen Thuy Thuong, Guy Thwaites, Louise Thwaites, Tran My Phuc, Tran Tan Thanh, Tran Thi Bich Ngoc, Tran Tinh Hien, H Rogier van Doorn, Jennifer Van Nuil, Vinh Chau, Vu Thi Ngoc Bich, Vu Thi Ty Hang, Sophie Yacoub.

***Acknowledgments.*** We acknowledge important contributions and guidelines from the following institutions and committees: Vietnam National Steering Committee for COVID-19 Prevention and Control, Ministry of Health, Ministry of Science and Technology, National Institute of Hygiene and Epidemiology and Pasteur Institutes. We also recognize Associate Prof. Bui The Duy, the director of the Rapid Response Information Team of the National Steering Committee for COVID-19 Response for his suggestions and support in the course of the investigation. We thank health-care workers from Provincial Centers of Disease Control, COVID-19 assigned hospitals and local authorities from provinces and cities in Vietnam for their work in case finding, contact tracing, disease control and prevention measures.

***Financial support.*** M. R., G. T., H. R. v. D., M. C. and the OUCRU COVID-19 research group are supported by the Wellcome Trust.

***Potential conflicts of interest.*** The authors: No reported conflicts of interest. All authors have submitted the ICMJE Form for Disclosure of Potential Conflicts of Interest.
