# Supplementary material for: The First 100 Days of Severe Acute Respiratory Syndrome Coronavirus 2 (SARS-CoV-2) Control in Vietnam
Source: Clin Infect Dis. 2020 Aug 1;72(9):e334–42. doi: 10.1093/cid/ciaa1130 (PMC7454342; doi:10.1093/cid/ciaa1130)
Supplement: ciaa1130_suppl_Supplementary_Appendix [file ciaa1130_suppl_supplementary_appendix.pdf]

The first 100 days of SARS-CoV-2 control in Vietnam

Pham Quang Thai[1,2], MD, Maia A Rabaa[3,4], PhD, Duong Huy Luong[5], MD, Dang Quang Tan[6], MD, Tran Dai Quang[6], MD, Ha-Linh Quach[1,7], MSc, Ngoc-Anh Hoang Thi[1,7], Phung Cong Dinh[8], Ngu Duy Nghia[1], MD, Tran Anh Tu[1], MD, La Ngoc Quang[9], MD, Tran My Phuc[4], MBA, Vinh Chau[4], MSc, Nguyen Cong Khanh[1], MD, Dang Duc Anh[1,*], MD, Tran Nhu Duong[1], MD, Guy Thwaites[3,4], MD, H Rogier van Doorn[3,4], MD, Marc Choisy[3,4], PhD, OUCRU COVID-19 Research Group†

[1]National Institute of Hygiene and Epidemiology, Vietnam
[2]School of Preventive Medicine and Public Health, Hanoi Medical University, Vietnam
[3]Centre for Tropical Medicine and Global Health, Nuffield Department of Medicine, University of Oxford, UK
[4]Oxford University Clinical Research Unit, Vietnam
[5]Medical Services Administration, Ministry of Health, Vietnam
[6]General Department of Preventive Medicine, Ministry of Health, Vietnam
[7]Research School of Population Health, Australian National University, Australia
[8]National Agency for Science and Technology Information, Ministry of Science and Technology, Vietnam
[9]Hanoi University of Public Health, Vietnam
†Members listed in the acknowledgments

***Corresponding author:** Professor Dang Duc Anh, Director, National Institute of Hygiene and Epidemiology, Hanoi, Vietnam. dda@nihe.org.vn

Contents **Page**

1 Data………………………………………… 2
1.1 Apple Maps Mobility Trends Reports………………… 2
1.2 Google Community Mobility Reports………………… 2
2 Methods………………………………………… 2
2.1 Origins of infection………………………………… 2
2.2 Serial intervals…………………………………… 3
2.3 Estimation of the reproduction number……………… 3
2.4 R packages used in the analysis…………………… 3
3 Results………………………………………… 3
Table S1……………………………………………… 4-9
Table S2……………………………………………… 10
Table S3……………………………………………… 10
Table S4……………………………………………… 10
4 References…………………………………………… 10

1 Data

1.1 Apple Maps Mobility Trends Reports

These data have been made available (https://www.apple.com/covid19/mobility) temporarily only, for the specific purpose of helping fighting against COVID-19[1]. Reports are published daily and reflect requests for directions in Apple Maps. This data is generated by counting the number of requests made to Apple Maps for directions, when moving by car or on foot. Data that is sent from users' devices to the Maps service is associated with random, rotating identifiers so Apple doesn't have a profile of the user's movements and searches. The data show a relative volume of directions requests in Vietnam compared to a baseline volume on January 13th, 2020. Day are defined midnight-to-midnight, UTC-8. Relative volume varies from week to week, consistent with normal, seasonal usage of Apple Maps (e.g. the lunar New Year holiday). Day of week effects (week-end versus week days) are thus important to normalize when reading these data. Data that is sent from users' devices to the Maps service is associated with random, rotating identifiers so Apple doesn't have a profile of individual movements and searches. Apple Maps has no demographic information about our users, so we can't make any statements about the representativeness of usage against the overall population.

1.2 Google Community Mobility Reports

These data have been made available (https://www.google.com/covid19/mobility) temporarily only, for the specific purpose of helping fighting against COVID-19[2]. These reports aim to provide insights into what has changed in response to policies aimed at combating COVID-19. The reports chart movement trends over time, across different categories of places such as **retail and recreation** (restaurants, cafes, shopping centers, theme parks, museums, libraries, and movie theaters), **groceries and pharmacies** (grocery markets, food warehouses, farmers markets, specialty food shops, drug stores, and pharmacies), **parks** (local parks, national parks, public beaches, marinas, dog parks, plazas, and public gardens), **transit stations** (public transport hubs such as subway, bus, and train stations), **workplaces**, and **residential**. No personally identifiable information, such as an individual's location, contacts or movement, is made available at any point. Insights in these reports are created with aggregated, anonymized sets of data from users who have turned on the Location History setting, which is off by default. Artificial noise is also added in order to prevent the identification of any individual person. The baseline is the median value, for the corresponding day of the week, during the 5-week period Jan 3rd – Feb 6th, 2020.

2 Methods

2.1 Origins of infection

Imported cases (noted G0 in the text) are all those who came back from an international trip. Among them, some were positive upon arrival, others became positive during the quarantine upon arrival and, before the systematic quarantine was put in place on March 21st, some others became sick and positive at home, in absence of any community transmission documented around them. All the cases who did not come back from an international trip were declared community-acquired. Among them, contact tracing allowed to

distinguish those (that we called G1) having been into contact with a G0 from the other ones (that we called G2+).

2.2 Serial intervals

A normal distribution was fitting by maximum likelihood to the values of serial intervals. The parameters (mean and standard deviation) estimates and their variance-covariance matrix were fed into a multinormal distribution in order to generate 10 000 Monte Carlo simulations of the serial interval distribution. We used these 10 000 simulated distributions to generate (i) the 95% confidence interval of the distribution of the serial intervals and (ii) the estimate, and its 95% confidence interval, of the proportion of distribution of the serial intervals that is below zero.

2.3 Estimation of the reproduction number

The reproduction number was estimated statistically from incidence data and the distribution of serial intervals as described and implemented in Cori 2019[8]. Essentially, the method consists in probabilistically reconstructing the transmission trees. A major advantage of the method is that it allows to compute the effective reproduction number with confidence interval of each time step chosen for the computation. A potential drawback is that it currently does not account for the fact that the distribution of the serial interval can be modified consecutively to interventions such as contact tracing.

2.4 R packages used in the analysis

All analyses were performed in R 4.0.0[3] with the following packages:

- fitdistrplus v. 1.0-14[4] for fitting the normal distribution to serial intervals.
- mvtnorm v. 1.1-0[5] for the multinormal distribution used in the Monte Carlo simulations.
- car v. 3.0-7[6] for the Type-II analyses of deviance in order to correct for potential confounding effects.
- incidence v. 1.7.1[7] to generate weekly incidence data from line listing. These weekly incidence data are then used to estimate weekly reproduction numbers.
- EpiEstim v. 2.2-1[8] to estimate the reproduction numbers by week.

3. Results

An extended version of **Table 1** is shown in **Table S1**.

Table S1. The timing and nature of major Government-led control measures, including international border control, internal control, Ministry of Health-led communications, and enhancement of diagnostic capability. The colors shown in the phase column indicate the intensity of control measures taken over different periods (white, initial; light yellow, early; light orange, intermediate; orange, pre-epidemic; brown, epidemic/lockdown; dark orange, post-lockdown), and correspond to the colors used in Table 1 and Figure 1 in the main text.

**Control measures at international borders**			
**Phase**	**Date**	**Event**	**Country of Origin**
	January 3, 2020	Deputy Director of the Preventive Medicine Department (Ministry of Health), signed a letter to direct the strengthening of quarantine at border gates, prevention of epidemics and the risk of novel viruses being transmitted into Vietnam	
	January 22, 2020	Monitoring of body temperature and health status at border gates, health facilities, and in communities, especially for passengers on arrival from Wuhan City, Hubei Province, China, and the affected region; early detection, isolation and timely handling of suspected cases of disease, preventing the disease from spreading to the community; monitor of all people exposed to a case within 14 days from the last exposure	China
	January 28, 2020	Suspension of all flights from epidemic areas in China except for special cases with the consent of the Prime Minister; temporary halt issuing of tourist visas to foreigners who have been in China in the past 2 weeks, except for official duty visas in special cases. Stop entry and exit with border laissez-passers for tourism purposes; border crossings at Vietnam-China border consolidated	China
	February 1, 2020	All flights between Vietnam and China suspended	China
	February 2, 2020	Halting of visa issuance, 14-day mandatory quarantine for all travelers who have come from or transited through COVID-19 affected areas in China	China
	February 5, 2020	Ban on entry from 31 affected provinces in China	China
	February 23, 2020	Medical declarations required for all incoming visitors from or transiting through Korea; all flights from infected zones diverted to secondary airports outside of HCMC and Hanoi, with all individuals coming from or passing through Daegu and North Gyeongsang, South Korea isolated in a centralized isolation area for 14 days upon entry; all entrants from Republic of Korea after February 8, 2020 to be identified for medical surveillance, monitoring and isolation	Republic of Korea
	February 25, 2020	Flights from infected zones redirected to secondary airports; inform travelers that they will be placed under centralized quarantine for 14 days when entering Vietnam	Republic of Korea
	February 28, 2020	Mandatory 14-day quarantine for all entering travelers	Korea, Iran, Italy
	February 29, 2020	Suspension of visa issuance	Republic of Korea
	March 3, 2020	Suspension of visa issuance	Italy

	March 6, 2020	Compulsory health declarations required for all passengers from Cambodia and European Union countries (EU)	Cambodia, EU
	March 7, 2020	Compulsory health declarations required for all passengers entering Vietnam from 06:00	Global
	March 15, 2020	Suspension of visa issuance	UK, EU
	March 18, 2020	Suspension of visa issuance to all foreigners for 30 days	Global
	March 21, 2020	Mandatory 14-day quarantine at centralized facilities for all arriving travelers, regardless of origin; by this date, nearly 300 000 VNmese citizens have returned from abroad, 100 000 from COVID-19 affected areas	Global
	March 21, 2020	Vietnam Airlines suspends routes	Singapore, Thailand, Indonesia, Laos, Myanmar
	March 22, 2020	Suspension of entry to all foreigners (except for diplomatic and official purposes); drastic cuts to incoming flights to avoid overloading the quarantine system	Global
	March 23, 2020	Vietnam Airlines suspends routes	UK, Japan
	March 27, 2020	All individuals entering the country from March 8 onward required to declare and update their health status to aid surveillance; strict control of all entrants by road, by sea and by air, especially the shared borders with Laos and Cambodia	Global, Cambodia and Laos
	April 1, 2020	Closure of main and auxiliary border gates	Cambodia and Laos

Internal control measures

Phase	Date	Event
	January 20, 2020	22 hospitals chosen for the treatment of suspected cases of nCoV
	January 22, 2020	Monitoring of body temperature and health status at border gates, health facilities, and in communities, especially for passengers on arrival from Wuhan City, Hubei Province, China, and the affected region; early detection, isolation and timely handling of suspected cases of disease, preventing the disease from spreading to the community; monitor of all people exposed to a case within 14 days from the last exposure
	January 30 to May 4, 2020	All schools and universities closed following lunar New Year holiday
	Late January - early February	Field hospitals and quarantine centers established in major cities and near border crossings
	February 2, 2020	MoH calls for review and standardization of nCoV testing
	February 6, 2020	Announcement that the Vietnam Social Insurance (VSI) health fund will cover 2019-nCoV tests and related treatment.
	February 12, 2020	Commune of 10 600 people in isolation after at least 10 cases of the novel coronavirus have been confirmed in Vinh Phuc province, north of Hanoi
	February 16, 2020	Joint effort to accelerate domestic production and supply of medical masks, protective uniforms and preventive medical supplies
	March 10, 2020	Nationwide Vietnam Health Declaration must be completed for all citizens and foreigners in the country

	March 13, 2020	Non-emergency travel abroad discouraged, domestic travel by groups to be limited in quantity, time, and activities while traveling	
	March 14, 2020	Verification of citizens of European countries entering Vietnam within the previous 14 days (from 13/3/2020 and earlier), request of compulsory electronic health declaration if declaration was not made upon entry into Vietnam	
	March 16, 2020	Enforcement of mask wearing at public places with possible crowds such as supermarkets, airports, stations, public transportation, etc.	
	March 20, 2020	Crowds over 50 people discouraged; entertainment services closed	
	March 21, 2020	Mandatory Health Declaration required for passengers on domestic flights and trains; suspension of in-person religious services	
	March 24 to April 23, 2020	HCMC: Ho Chi Minh City People's Committee issues a document to suspend operation of amusement parks, restaurants and beer clubs; catering businesses (with a capacity of 30 people or more), billiard clubs, gyms, spas, hair salons	
	March 28, 2020	Medical isolation measures required for all medical personnel interacting with COVID-19 patients and their family members; 14-day isolation, starting from 00:00 on March 28, 2020 to 00:00 on April 11, 2020	
	March 28 - April 15, 2020	Drastic reduction in domestic flights, decreasing total number of seats on flights by 90%	
	March 28, 2020	Gatherings of >20 people strongly discouraged; gatherings of >10 people or more outside the workplace, school, hospital prohibited; individuals must maintain a minimum distance of 2m in public places; provincial/city governments determine essential businesses that may remain open; temporary suspension or reorganization of public transport activities to restrict travel and social contact	
	April 1, 2020	Declaration of COVID-19 epidemic in Vietnam	
	April 1 to April 22, 2020	Implementation of social isolation for 15 days (extended) on a national scale according to the principle that families are separated from families, villages from villages, communes from communes, districts from districts and provinces from provinces. Country-wide lockdown implemented, including closure of all shops except those selling food, medications and fuel; mandatory mask-wearing in public; banning of public gatherings of >2 people; non-essential movement outside of residence discouraged; public transportation and taxi services halted	
	April 16 to April 22, 2020	Gradual increase in domestic flights	
	April 23, 2020	Lockdown measures relaxed, some non-essential businesses such as bars, spas, karaoke bars, and massage parlors remain closed; increased frequency of domestic flights	
	May 4 to May 11, 2020	Staggered reopening of schools and universities	

Ministry of Health-led Communications

Phase	Date	Event	Responsible party
	January 9, 2020 onward	Dissemination of information to people, communities and healthcare workers, advising people on the disease situation in China and to maintain calm and proactively prevent winter-spring epidemic diseases according to the recommendations of the Ministry of Health. Promotion of disease surveillance at border gates, monitoring of patients with unexplained severe pneumonia at health care facilities, paying special attention to cases with history related to epidemic areas	Public Health Emergency Response Center, various news outlets

	January 20, 2020	Updated information to be provided every 2 hours on ncov.moh.gov.vn and ncov.vncdc.gov.vn	MoH and Department of Preventive Medicine websites
	January 27, 2020	Telephone hotline number announced to receive information and opinions on the epidemic situation at the nationwide level and to advise on personal disease prevention	Various news outlets, government websites
	February 2, 2020	Technology-based communication plan established to inform population: SMS to all mobile subscribers; videos and infographics disseminated through mass media and on social networks and digital platforms such as Facebook, Zalo, YouTube, Lotus, etc.; uniform use of hashtag **#ICT_anti_nCoV**	Government-wide
	February 3, 2020	Those in contact with infected/suspected COVID-19 case must notify health authorities, wear a mask, and limit contact with others. All people should wash hands and other items often with soap, wear masks in public places and on public transport. Travel to China restricted. MoH web portal established for population monitoring	MoH SMS
	February 4, 2020	Encourage nationwide efforts against nCoV to comply with regulations and recommendations of the health sector. Discourage the distribution of false information and profiteering	MoH SMS
	February 5, 2020	Refocusing of communications on disease prevention, guidance on community sanitation, public places, experience on epidemic prevention of previous epidemics, setting good examples, good work in epidemic prevention, silent sacrifices of health workers, individual roles and responsibilities in preventing disease in the community; reduce focus on warnings about disease risk and avoiding sensational news	National Steering Committee decision
	February 6, 2020	Recommendation for use of medical masks when in contact with an infected/suspected nCoV case or someone with symptoms of respiratory disease, self-monitoring and isolation at home. Encourage the use of cloth masks for all healthy individuals when in crowded areas or on public transport. Reminder that hands washing and personal hygiene remain important measures to prevent the spread of disease	MoH SMS
	February 8, 2020	Vietnam Health App and website launched by MoH to provide information on COVID-19 and disease prevention, aiming to provide accurate, comprehensive, diverse and supportive information sources for the people, as well as healthcare workers	Various news outlets, government websites
	February 11, 2020	Encourage hand washing with soap and use of alcohol-based hand sanitizers when soap and water are not available	MoH SMS
	February 13, 2020	Guidance on medical isolation at home and in places of residence provided from vncdc.gov.vn	MoH SMS
	February 14, 2020	Develop notice of the 14-day isolation period with COVID-19 (nCoV); messages on treatment hierachy for COVID-19 (nCoV); coordinate with Vietnam Television (VTV) to prepare programs on "World Panorama"	MoH decision
	February 14, 2020	Release of an iOS and Android app for health information about nCoV	MoH SMS
	February 19, 2020	Encourage people with signs of COVID-19 acute respiratory infection: fever, cough, shortness of breath and close contact with case or suspicion of infection to contact appropriate medical facilities, provide medical and contact histories, and go to the nearest district hospital for examination and treatment if indicated by authorities. Reminder that the rapid response teams of the Ministry of Health are always available, supporting district hospitals in the work of examination, diagnosis and treatment for patients	MoH SMS
	February 23, 2020	Release of pop song, Ghen Cô Vy (English: Jealous Coronavirus), to promote handwashing, social distancing, not touching one's face, and keeping their surroundings clean	MoH and Vietnam

			National Institute of Occupational and Environmental Health (VNNIOSH)
	February 26, 2020	Recommendation issued for prevention and control of COVID-19 (nCoV) for trade centers, supermarkets, markets, restaurants, hotels, parks and tourist areas	MoH recommendation
	March to April 2020	Regular assurances of availability of food, medicines and other necessities, recommendations for online shopping to limit crowding in public places, requests to fill out universal health declarations, denial of unfounded rumors, communication of regulations on public gatherings, mask wearing, and unnecessary travel	SMS messages from local government authorities
	March 2, 2020	MoH coordinates with Vietnam Television (VTV 24) to develop a health news and broadcast at 11:30 noon daily on the situation of COVID-19 epidemic on VTV1	MoH decision
	March 7, 2020	When entering Vietnam, an accurate and truthful medical declaration must be provided, either at the border gate or online from suckhoetoandan.vn	MoH SMS
	March 8, 2020	Indication of monitoring of cases and contacts, discouraging overreaction while safely implementing MoH guidelines for hygiene and safety	MoH SMS
	March 9, 2020	Encourage hand washing and proper hygiene, limiting contact with those who are ill, proper mask wearing	MoH SMS
	March 10, 2020	Personal hygiene recommendations	MoH SMS
	March 12, 2020	Encourage those with signs of COVID-19 acute respiratory infection: fever, cough, shortness of breath and close contact with case or suspicion of infection to contact appropriate medical facilities, provide medical and contact histories, and go to the nearest district hospital for examination and treatment if indicated by authorities. Reminder that the rapid response teams of the Ministry of Health are always available, supporting district hospitals in the work of examination, diagnosis and treatment for patients	MoH SMS
	March 13, 2020	Guidelines for isolation at home provided at ncov.moh.gov.vn	MoH SMS
	March 14, 2020	Reminder of the MoH telephone hotline and Vietnam Health app for up-to-date information on the COVID-19 epidemic	MoH SMS
	March 19, 2020	Mandatory use of the Hanoi Smart City app to monitor the health and movement of recovered confirmed cases, suspected cases, and people under quarantine	Various news outlets, government websites
	March 22, 2020	Instructions for all individuals entering Vietnam from 1 March 2020 to make an online medical declaration, self-separate at home (if not yet in quarantine) for 14 days after return to Vietnam. Reminder for all to wear a mask and wash hands frequently	MoH SMS
	March 22, 2020	Recommendation that people over 60 stay at home and limit contact with others. Recommendation that everyone wear a mask when outside of the home and practice good hygiene	Office of the Prime Minister SMS
	March 29, 2020	Strong recommendations on mask wearing, hygiene, minimization of travel and social contact	MoH SMS
	March 31, 2020	Strong recommendations on mask wearing, hygiene, minimization of travel and social contact; reminder of mandatory medical declarations on NCOVI application NCOVI application	MoH SMS

	April 18, 2020	Release of Bluezone mobile application that uses BLE low-power Bluetooth positioning technology to identify and track and communicate with F1 and F2 contacts when positive cases are detected	MoH and Ministry of Information and Communications

Testing capacity

Phase	Date	Cumulative number of PCR tests administered	Total number of testing sites nationwide
	January 23, 2020	10	2
	February 1, 2020	237	5
	February 16, 2020	984	6
	March 1, 2020	2,370	8
	March 16, 2020	14,291	16
	April 1, 2020	83,194	38
	April 16, 2020	206,464	52
	May 1, 2020	266,122	63

Table S2. Analysis of deviance table (type-II tests) for Figure 3B explaining the duration of hospitalization with a gamma regression. LR Chisq: chi-squared statistic, Df: degrees of freedom, Pr(>Chisq): p-value of the type-II likelihood ratio test.

	LR Chisq	Df	Pr(>Chisq)
asymptomatic	18.9059	1	< 0.0001
age	1.4677	1	0.2257
imported	0.4023	1	0.5259
gender	0.0530	1	0.8180
nationality	2.4260	1	0.1193

Table S3. Analysis of deviance table (type-II tests) for Figure 3C explaining the proportion asymptomatic with a logistic regression. LR Chisq: chi-squared statistic, Df: degrees of freedom, Pr(>Chisq): p-value of the type-II likelihood ratio test.

	LR Chisq	Df	Pr(>Chisq)
age	0.1999	1	0.6548
imported	3.0757	1	0.0795
gender	0.0915	1	0.7623
nationality	0.3049	1	0.5808

Table S4. Contingency table of the number of symptomatic and imported cases. The total number of cases is 270.

	imported	locally transmitted	totals
asymptomatic	71	49	120
symptomatic	92	58	150
totals	163	107	270

4. References

1 Apple LLC. Apple COVID-19 Reports on Mobility Trends. https://www.apple.com/covid19/mobility (accessed May 7, 2020).

2 Google LLC. Google COVID-19 Community Mobility Reports. https://www.google.com/covid19/mobility/ (accessed May 7, 2020).

3 R Core Team. R: A Language and Environment for Statistical Computing. Vienna, Austria: R Foundation for Statistical Computing, 2020 https://www.R-project.org/.

4 Delignette-Muller ML, Dutang C. fitdistrplus: An R Package for Fitting Distributions. *Journal of Statistical Software* 2015; **64**: 1–34.

5 Genz A, Bretz F, Miwa T, *et al.* mvtnorm: Multivariate Normal and t Distributions. 2020 https://CRAN.R-project.org/package=mvtnorm.

6 Fox J, Weisberg S. An R Companion to Applied Regression, Third. Thousand Oaks CA: Sage, 2019 https://socialsciences.mcmaster.ca/jfox/Books/Companion/.

7 Kamvar ZN, Cai J, Pulliam JRC, Schumacher J, Jombart T. Epidemic curves made easy using the R

package incidence [version 1; referees: awaiting peer review]. *F1000Research* 2019; **8**. https://doi.org/10.12688/f1000research.18002.1.
8 Cori A. EpiEstim: Estimate Time Varying Reproduction Numbers from Epidemic Curves. 2019 https://CRAN.R-project.org/package=EpiEstim.
